# Identification of N6-methylandenosine related LncRNAs biomarkers associated with the overall survival of osteosarcoma

**DOI:** 10.1186/s12885-021-09011-z

**Published:** 2021-12-01

**Authors:** Pei Zhang, Keteng Xu, Jingcheng Wang, Jiale Zhang, Huahong Quan

**Affiliations:** 1 Department of Orthopedics, The Second Xiangya Hospital of Central South University, Changsha, 410011 Hunan China; 2 Department of Joint surgery, Huangshan City People’s Hospital, Huangshan, Anhui China; 3 Department of Orthopedics, Clinical Medical College, Yangzhou University, Northern Jiangsu People’s Hospital, Yangzhou, China; 4 Department of Graduate, Dalian Medical University, Dalian, 116044 Liaoning China

**Keywords:** Osteosarcoma, N6-methylandenosine, Bioinformatics

## Abstract

**Purpose:**

Osteosarcoma (OS) is a differentiation disease caused by the genetic and epigenetic differentiation of mesenchymal stem cells into osteoblasts. OS is a common, highly malignant tumor in children and adolescents. Fifteen to 20 % of the patients find distant metastases at their first visit. The purpose of our study was to identify biomarkers for tracking the prognosis and treatment of OS to improve the survival rate of patients.

**Materials and methods:**

In this study, which was based on Therapeutically Applicable Research to Generate Effective Treatments (TARGET), we searched for m6A related lncRNAs in OS. We constructed a network between lncRNA and m6A, and built an OS prognostic risk model.

**Results:**

We identified 14,581 lncRNAs by using the dataset from TARGET. We obtained 111 m6A-related lncRNAs through a Pearson correlation analysis. A network was built between lncRNA and m6A genes. Eight m6A-related lncRNAs associated with survival were identified through a univariate Cox analysis. A selection operator (LASSO) Cox regression was used to construct a prognostic risk model with six genes (RP11-286E11.1, LINC01426, AC010127.3, DLGAP1-AS2, RP4-657D16.3, AC002398.11) obtained through least absolute shrinkage.

We also discovered upregulated levels of DLGAP1-AS2 and m6A methylation in osteosarcoma tissues/cells compared with normal tissues/osteoblasts cells.

**Conclusion:**

We constructed a risk score prognosis model of m6A-related lncRNAs (RP11-286E11.1, LINC01426, AC010127.3, DLGAP1-AS2, RP4-657D16.3, AC002398.11) using the dataset downloaded from TRAGET. We verified the value of the model by dividing all samples into test groups and training groups. However, the role of m6A-related lncRNAs in osteosarcoma needs to be further researched by cell and in vivo studies.

## Background

Osteosarcoma (OS), a disease that mainly affects children and adolescents, is a common malignant tumor that occurs in the mesenchymal tissue of the metaphysis of long bones [1–3]. Although extensive surgical resection combined with chemotherapy and radiotherapy has achieved certain good results, about 40–50% of patients experience lung metastasis [4, 5]. The five-year survival rate of the patients with lung metastases is only 28%. Thus, it is important to develop a novel therapeutic approach to effectively treatOS.

More than 60% of all RNA modifications are RNA methylation. N6-methyladenosine (m6A) is the most common form of modification of human messenger RNA (mRNA) and long non-coding RNA (lncRNA) [6, 7]. M6A modification mainly occurs on the adenine of the RRACH sequence, and its function is mainly related to writers, readers, and erasers. Writers (RBM15B, METTL3, METTL14, METTL16, RBM15, WTAP, VIRMA [KIA1499], and ZC3H13) are methyltransferases [8, 9]. The eraser is a demethylase that can reverse methylation and includes FTO and ALKBH5. The reader is signal transducers, with m6A binding protein that can bind to M6A, including FMR1, HNRNPC, HNRNPA2B1, IGFBP1, IGFBP2, IGFBP3, LRPPRC, RBMX, YTHDC1, YTHDC2, YTHDF1, YTHDF2, and YTHDF3. Accumulating evidence has shown that m6A modification plays an important role in tumorigenesis and tumor progression, such as hepatocellular carcinoma, lung cancer, and leukemia [10–12]. Wang et al. revealed that METTL3 promotes tumorigenesis by up-regulating m6A modification of APC [13]. Qu et al. found that HBx and ALKBH5 promote hepatocellular carcinogenesis through a positive feedback loop [14]. Cao et al. reported that IGF2BP2 can regulate insulin sensitivity and glucose metabolism to mediate cancer pathogenesis [11]. Additionally, ALKBH5 has been shown to negatively regulate the osteogenic differentiation of mesenchymal stem cells through PRMT6 [15].

LncRNAs, a class of RNA transcripts, are greater than 200 nucleotides in length [16]. Although they generally cannot encode proteins or peptides, lncRNAs play an important role in cell activation, proliferation, differentiation, apoptosis and metabolism by regulating gene expression and function [16]. Recent research had shown that dysregulated lncRNA plays an important role in many diseases, such as tumor, cardiovascular diseases, and metabolic disorders [17, 18]. Dysregulated lncRNA is closely associated with tumor growth and metastasis observed in cancers such as breast, lung, liver, and colorectal cancers [19–21]. Some studies have suggested that lncRNAs may potentially serve as biomarkers and targets in diagnosing and treating tumors.

Bioinformatics is a new field of biological research, which involves the processing and analysis of biological data using mathematical, statistical and computational methods. Due to the large amount of data generated by new technologies such as genome sequencing and microarray chip technology, the traditional gene-by-gene method is not enough to meet the growth and demands of biological research. Therefore, bioinformatics is a valuable way to expand biological insight and promote the development of new therapeutic approaches. We searched for m6A -related lncRNAs, based on Therapeutically Applicable Research to Generate Effective Treatments (TARGET, https://ocg.cancer.gov/programs/target). Using bioinformatic and statistical analysis methods, we constructed a prognostic risk model to identify biomarkers related to OS prognosis and treatment to improve the survival rate of patients.

## Methods

### Data collection and data processing

We downloaded the OS gene expression profiles, and the corresponding clinical data from the TARGET database, which comprised a total of 84 OS samples. LncRNAs were identified using the Ensemble IDs of the genes. All lncRNAs were extracted base on the perl. Expression matrixes of 23 m6A genes were extracted from previous publications, including the expression data on writers (RBM15B, METTL3, METTL14, METTL16, RBM15, WTAP, VIRMA [KIA1499], and ZC3H13), readers (FMR1, HNRNPC, HNRNPA2B1, IGFBP1, IGFBP2, IGFBP3, LRPPRC, RBMX, YTHDC1, YTHDC2, YTHDF1, YTHDF2, and YTHDF3) and erasers (FTO and ALKBH5).

### Identification of m6A-related lncRNAs

Using a Pearson correlation analysis, we searched for m6A-related lncRNAs in each gene (with the | Pearson R| > 0.3 and *P* < 0.01). The interaction betweenlncRNA and m6A was visualized through the igraph R package. We then used a univariate Cox regression analysis to mine the prognostic m6A-related lncRNAs (pFilter = 0.05).

### Identification of cluster based on m6A-related lncRNAs and gene set enrichment analysis

The data matrix of m6A-related lncRNAs was inputted to ConsensusClusterPlus. Stability evidence for a given number of groups (k) and cluster assignments was outputted. We set the number of clusters, k, from 2 to 10, and found the ideal k from it. A heatmap was generated using the ‘pheatmap’ R packages. A Kaplan–Meier (KM) survival curve was drawn to assess prognoses between the clusters. Gene Set Enrichment Analysis has been widely applied to perform KEGG (Kyoto Encyclopedia of Genes and Genomes) and GO (Gene Ontology) analysis. The top ten pathways were shown.

### Construction of prognosis risk score model

Utilizing a least absolute shrinkage and selection operator (LASSO) regression analysis, all m6A-related lncRNAs were analyzed to select the most optimal prognostic biomarkers and to construct the risk score model. A LASSO regression analysis is a penalty regression method that reduces overfitting by simultaneously performing shrinkage and model selection. Some coefficients can be compressed to 0 based by constructing a penalty function, resulting in a more refined model. Therefore, the superiority of subset shrinkage is retained. In addition, the LASSO model can perform biased estimation of multicollinearity data processing and realize the selection of variables in the estimation. The ideal method to solve the multicollinearity problem in a regression analysis is a LASSO regression analysis.

### Validation of prognosis risk score model

In this study, we calculated the prognosis risk scores based on a formula as follows:

$$\mathrm{Risk}\kern0.17em \mathrm{score}=\sum {\mathrm{lncRNAs}}_{\mathrm{Cox}\kern0.24em \mathrm{coefficient}}\times {\mathrm{lncRNAs}}_{\mathrm{expression}\kern0.17em \mathrm{levels}}.$$

We divided all samples into low-risk groups and high-risk groups, using the median risk score as the cutoff value. To avoid random allocation bias, all OS patients were randomly grouped at a ratio of 0.5:0.5 into a training cohort and a test cohort. Using a KM survival analysis, and calculating the area under the ROC curve (AUC), and etc., we assessed the accuracy and efficiency of the risk score model. We performed the univariate and multivariate Cox survival analyses according to clinical factors such as sex, age, primary site of OS and metastasis to assess the predictive independence of the risk score model. In addition, KM survival analysis was applied to confirm the effectiveness of the risk score model for different clinical features.

#### RNA m6A quantification

The m6A RNA methylation assay kit (ab185912; Abcam, UK) was utilized to measure the m6A content. Briefly, extracted RNAs were coated on each well, followed by adding capture antibody and detection antibody. Finally, the m6A levels were detected by measuring the absorbance at 450 nm using a microplate reader.

#### RNA isolation and qRT-PCR

Total RNA was isolated using RNeasy Mini Kit (Qiagen, Germany), and reversely transcribed according to the standard of cDNA Synthesis Kit (Vazyme, China). Then, qRT-PCR was performed using SYBR Kit (Vazyme, China) to evaluate the mRNA levels of DLGAP1-AS2 and GAPDH according to the supplier’s instructions.

## Results

### Flow chart of analysis

**Fig. 1:**
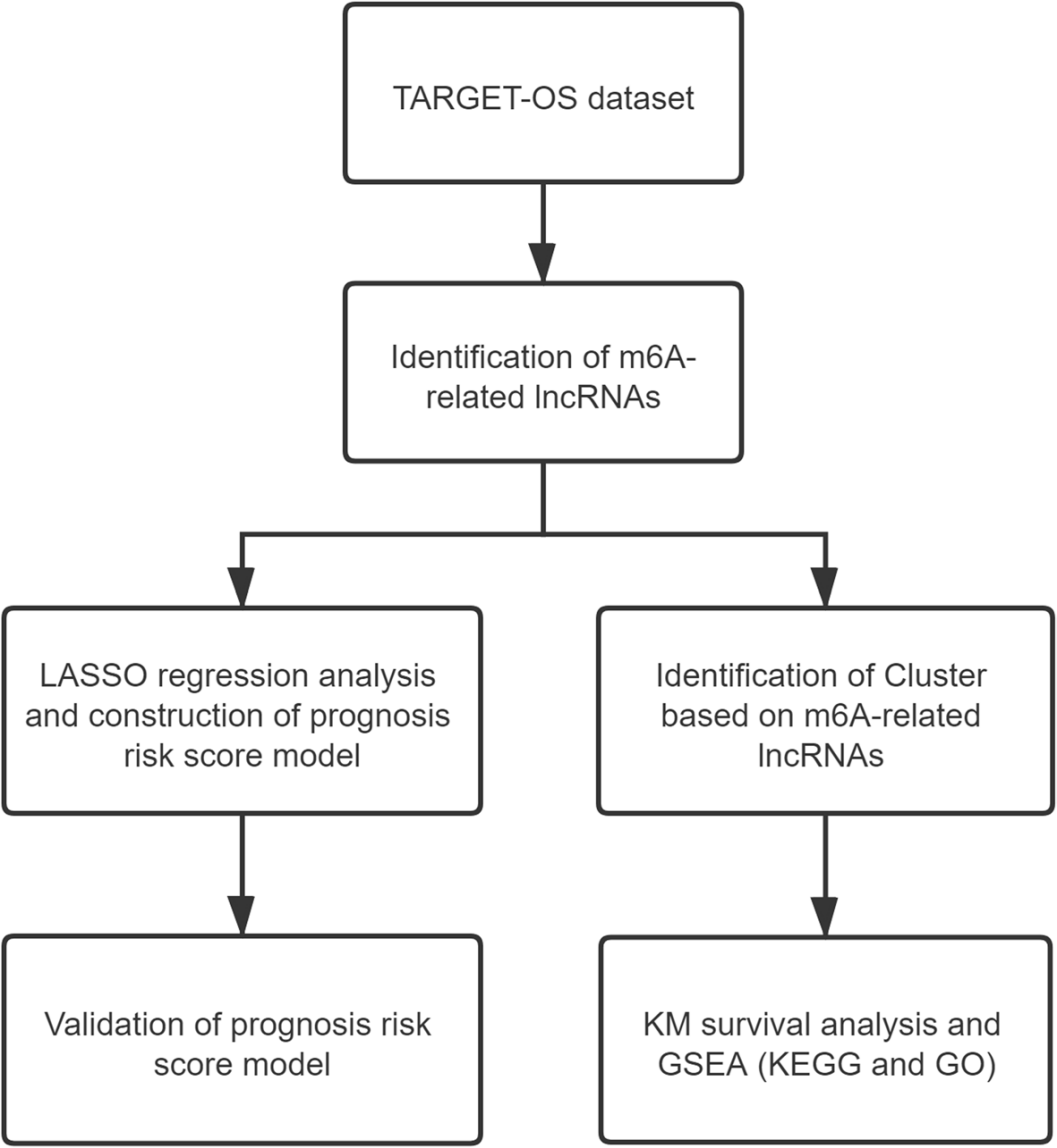
Study flow chart

We designed a protocol to analyze m6A-related lncRNAs and constructed the prognosis risk score model (Fig. 1).

### Screening m6A-related lncRNAs in OS patients

**Fig. 2:**
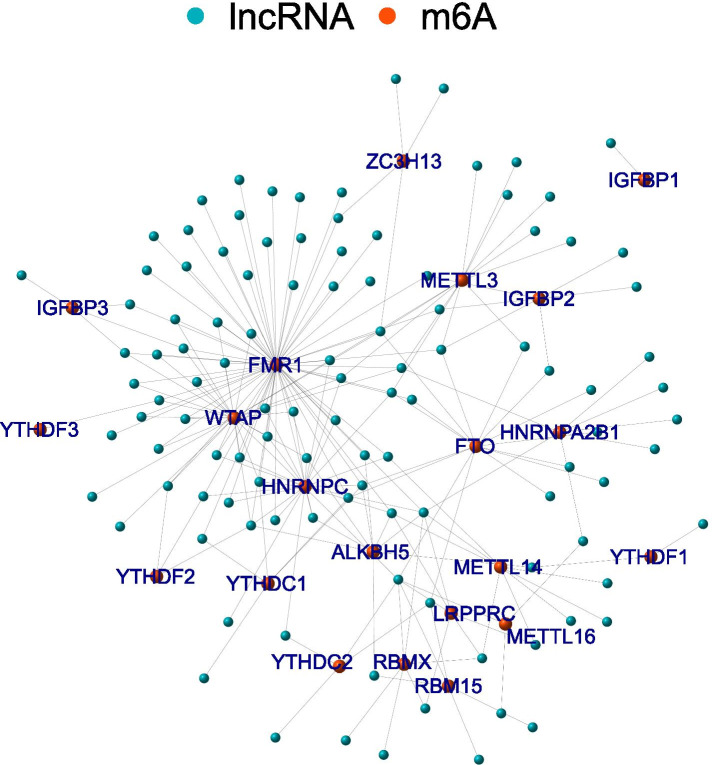
The lncRNA–m6A networks

First, we identified 14,581 lncRNAs by using the OS dataset from TARGET. We then extracted the expression matrixes of 23 m6A-related lncRNA. A lncRNA was defined as an m6A-related lncRNA with an expression value was correlated with one or more of the 23 m6A-related genes (| Pearson R| > 0.3 and *p* < 0.01). We obtained 111 m6A-related lncRNAs through Pearson correlation analysis. The network was built between lncRNA and m6A (Fig. 2).

### Identification of clusters according to m6A-related lncRNAs

**Fig. 3:**
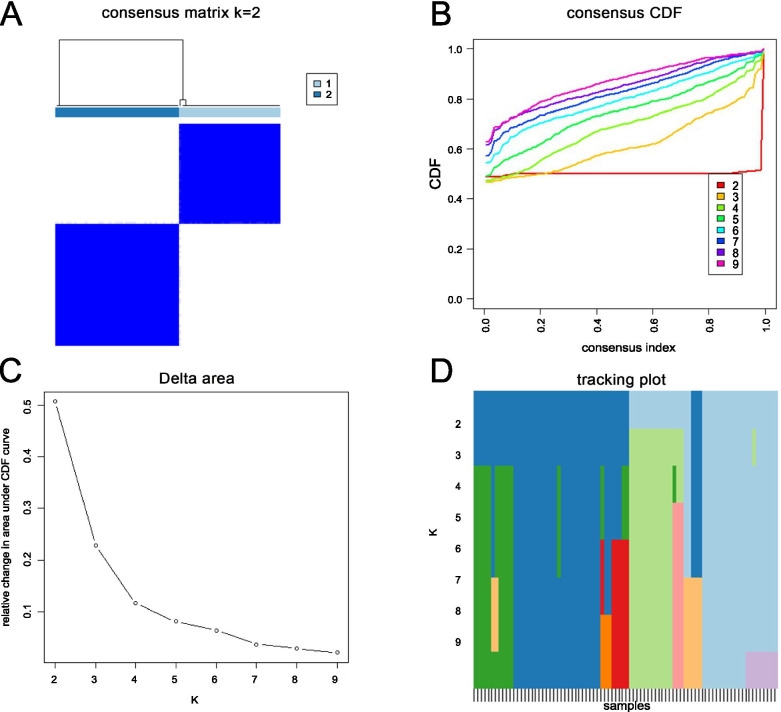
**A** Consensus clustering matrix for k = 2. **B** Cumulative distribution function for k = 2 to 9. **C** Relative change in the area under the CDF curve for k = 2 to 9. **D** Tracking plot for k = 2 to 9

Eight m6A-related lncRNAs were identified using univariate Cox analysis (RP11-799D4.4, RP11-286E11.1, LINC01426, AC010127.3, DLGAP1-AS2, RP4-657D16.3, RP1-178F15.5, and AC002398.11). The results of the 8 m6A-related lncRNAs are shown in Table 1. According to the consensus clustering analysis, the optimal number of clusters was k = 2 (Fig. 3A, B, C, D). Compared with the Cluster 2 (C2) subtype, most genes were highly expressed in the Cluster 1 (C1) subtype.

**Table 1:** The Univariate Cox analysis result of the 8 m6A-related lncRNAs

Gene	HR	HR.95L	HR.95H	pvalue
RP11-799D4.4	0.9981	0.9966	0.9995	0.0087
RP11-286E11.1	1.0016	1.0000	1.0032	0.0489
LINC01426	0.9944	0.9900	0.9988	0.0128
AC010127.3	1.0165	1.0030	1.0302	0.0164
DLGAP1-AS2	1.0017	1.0001	1.0032	0.0328
RP4-657D16.3	0.9965	0.9932	0.9998	0.0372
RP1-178F15.5	1.0088	1.0003	1.0174	0.0423
AC002398.11	0.9993	0.9987	0.9999	0.0186

### Prognostic analysis and biological functional analysis of the m6A-related lncRNAs

**Fig. 4:**
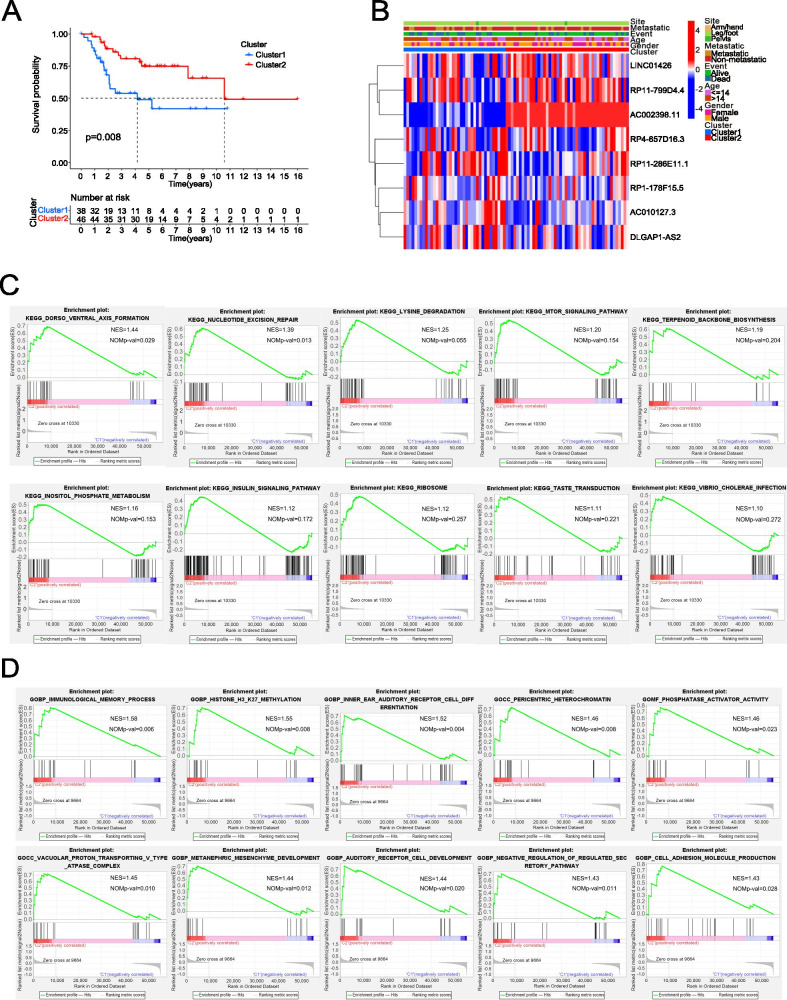
**A** KM survival curves of C1 and C2. **B** The heatmap of the relationships between the expression levels of 8 m6A-related lncRNAs and clinicopathological characteristics. **C** The outcome of KEGG analysis. **D** The outcome of GO analysis

The KM survival curve was drawn to compare the survival of the two molecular subtypes. In the results, the survival rate of C2 was significantly better than that of C1 (*P* < 0.05; Fig. 4A). A heatmap of the relationships between the expression levels of these eight m6A-related lncRNAs and clinicopathological characteristics were shown in Fig. 4B. GSEA was performed to analyze the KEGG and GO analysis in C1 and C2(Fig. 4C,D).

### Construction of the prognostic risk model

**Fig. 5:**
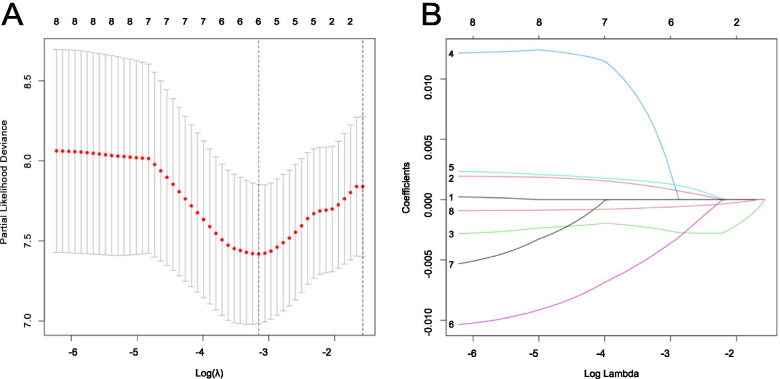
**A** The changing trajectory of each independent variable. **B** The confidence interval of each lambda

Using a LASSO-Cox regression analysis, we further selected appropriate m6A-related lncRNAs to maintain a high accuracy rate. We show the changing trajectory of each independent variable in Fig. 5A. The confidence interval of each lambda was shown in Fig. 5B. These figures showed that a total of six genes were selected as the target genes for subsequent analysis when the model reached the optimal value. The six m6A-related lncRNAs were obtained as the most suitable predictors for prognosis (RP11-286E11.1, LINC01426, AC010127.3, DLGAP1-AS2, RP4-657D16.3, AC002398.11). A risk scoring model to predict the prognosis of OS patients was constructed as follows: RP11-286E11.1 * 0.00097667835205502 + LINC01426 * (− 0.00245896608774788) + AC010127.3 * (0.00475697115852846) + DLGAP1-AS2 * (0.00138111340031212) + RP4-657D16.3 * (− 0.00419685380105118) + AC002398.11 * (− 0.000637455261090537).

### Evaluation of the risk model

**Fig. 6:**
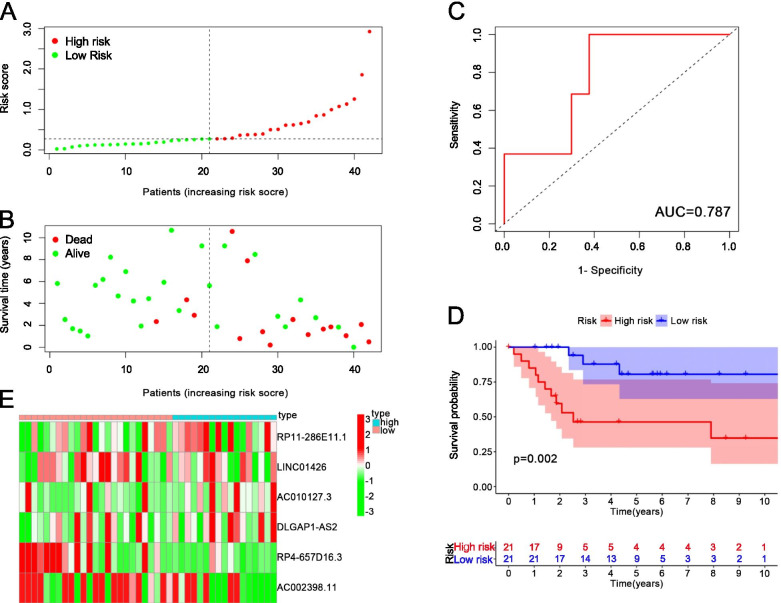
**A** The risk score distribution of the training cohort. **B** The survival time of the training cohort. **C** The AUC of the training cohort. **D** The KM survival curves of the training cohort. **E** The heatmap of the expression of m6A-related lncRNAs in the high-risk group and low-risk group

**Fig. 7:**
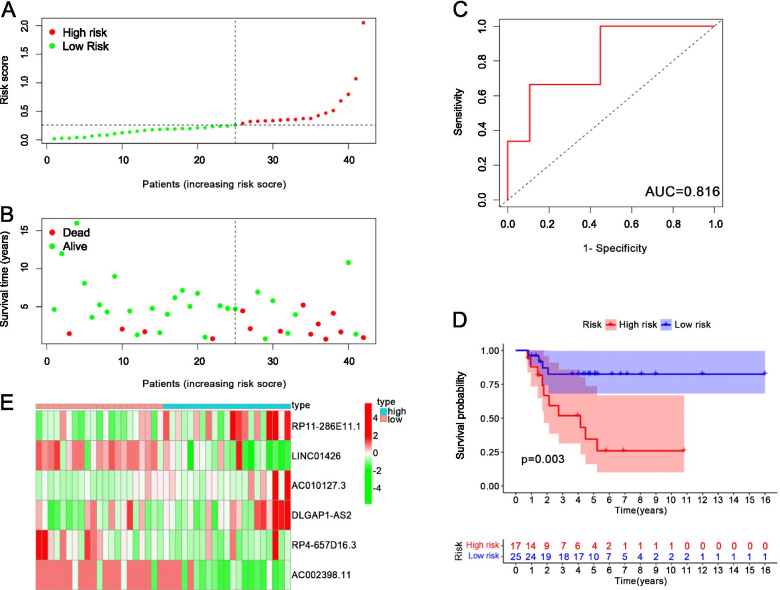
**A** The risk score distribution of the test cohort. **B** The survival time of the test cohort. **C** The AUC of the test cohort. **D** The KM survival curves of the test cohort. **E** The heatmap of the expression of m6A-related lncRNAs in the high-risk group and low-risk group

We randomly divided the 84 samples into two groups, 42 samples were used as the testing cohort samples, and the other 42 samples were used as the training cohort samples. The risk score distribution of the training cohort is shown in Fig. 6A, and the survival time of the training cohort is shown in Fig. 6B. The average area under the curve (AUC) of the training cohort reached 0.787 (Fig. 6C). The training cohort have 21 samples as high risk and 21 as low risk. There was a significant difference between high-risk samples and low-risk samples in the KM survival curves (*P* = 0.002, Fig. 6D). The heatmap showed the high-risk samples and low-risk samples had different m6A-related lncRNA expression levels (Fig. 6E). The risk score distribution of the test cohort is shown in Fig. 7A, and Fig. 7B shows the survival time of the test cohort. The AUC of test cohort was 0.816 (Fig. 7C). The test cohort had 17 high-risk samples and 25 low-risk samples.. There was a significant difference between high-risk samples and low-risk samples in the KM survival curves (*P* = 0.003, Fig. 7D). The heatmap shows that the high-risk samples and low-risk samples had different expression levels of m6A-related lncRNA (Fig. 7E).

### Prognostic analysis of the risk model and clinical features

**Fig. 8:**
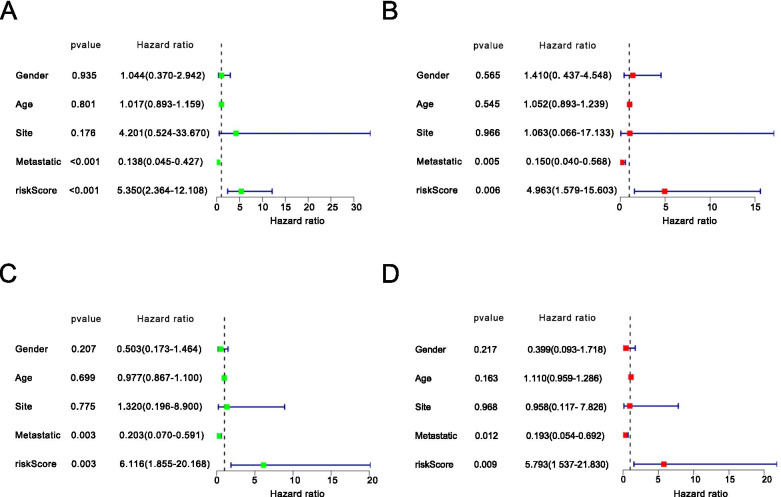
**A** Univariate Cox regression analyses of the training group. **B** Multivariate Cox regression analyses of the training group. **C** Univariate Cox regression analyses of the test group. **D** Multivariate Cox regression analyses of the test group

**Fig. 9:**
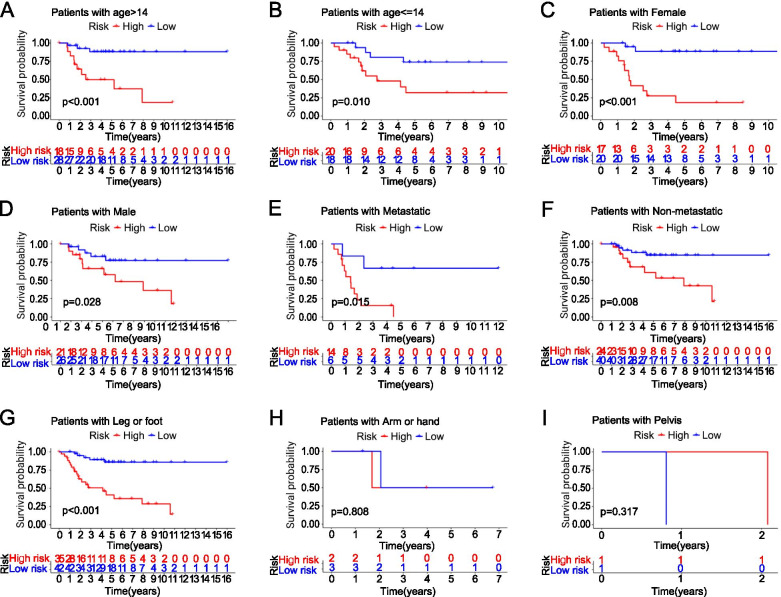
**A** The KM survival curves of age > 14. **B** The KM survival curves of age < =14. **C** The KM survival curves of female. **D** The KM survival curves of male. **E** The KM survival curves of Metastatic. **F** The KM survival curves of Non-metastatic. **G** The KM survival curves of Leg. **H** The KM survival curves of Arm. **I** The KM survival curves of Pelvis

Using a univariate and multivariate Cox regression analysis, we analyzed clinical variables, such as age, gender, site, and metastasis (Fig. 8A, B, C, D). The *P-*value of the univariate and multivariate Cox survival analysis in the training group and the test groupwas < 0.05. The results suggested that the model was an independent risk factor of survival for OS patients. We have drawn the KM survival curves according to the clinical characteristics to compare the survival of the high-risk group and low-risk group. In the dataset, 38 patients were 14 years of age or younger, and 46 patients older than 14 years. In the group of age over 14, there are 18 patients as high risk and 28 patients as low risk (*P* < 0.001, Fig. 9A). In the group 14 years of age or younger, 20 patients were high risk and 18 patients as low risk (*P* = 0.010, Fig. 9B). Of the 84 OS patients, there were 37 women and 47 men. Seventeen women were classified into the high-risk group and 20 women were classified into the low-risk group (*P* < 0.001, Fig. 9C). Twenty-one men were classified into the high-risk group and 26 men were classified into the low-risk group (*P* = 0.028, Fig. 9D). Twenty patients had metastases, while the remaining 64 patients had no metastases. Among the patients with OS metastasis, 14 patients were in the high-risk group and 6 patients were in the low-risk group (*P* = 0.015, Fig. 9E). Among patients without metastasis, 24 patients were in the high-risk group and 40 patients s were in the low-risk group (*P* = 0.008, Fig. 9F). The primary OS site was the leg or foot in 77 patients, the arm or hand in 5 patients, and the pelvis in 2 patients., s. In the group of patients with the leg as the primary OS site, 35 patients s were high risk and 42 patientswere low risk (*P* < 0.001, Fig. 9G). Because the number of patients with the arm (Fig. 9H) or the pelvis (Fig. 9I) as the primary OS site was too small, the results were meaningless.

**Fig. 10:**
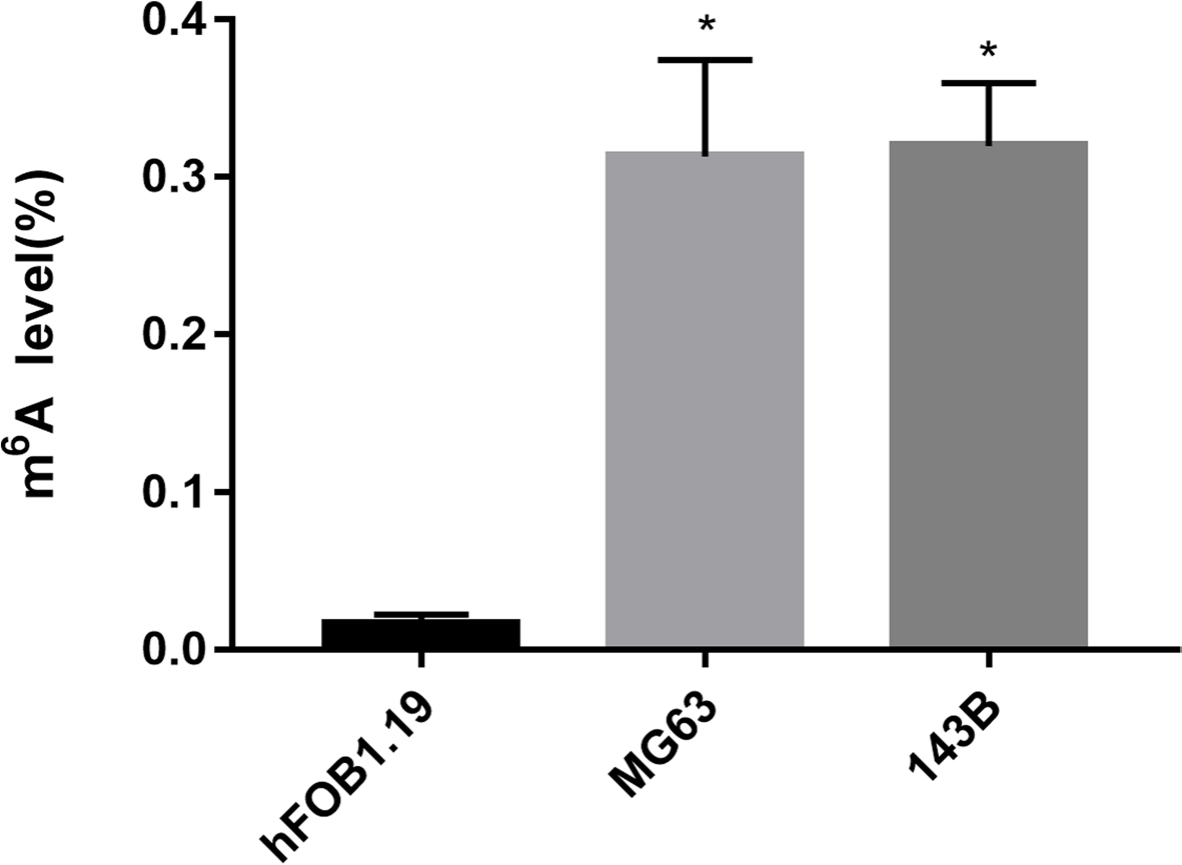
The overall m6A level of RNA in human osteosarcoma cell line (MG63,143B) and human osteoblast cell line(hFOB1.19).*P < 0.05 versus hFOB1.19

**Fig. 11:**
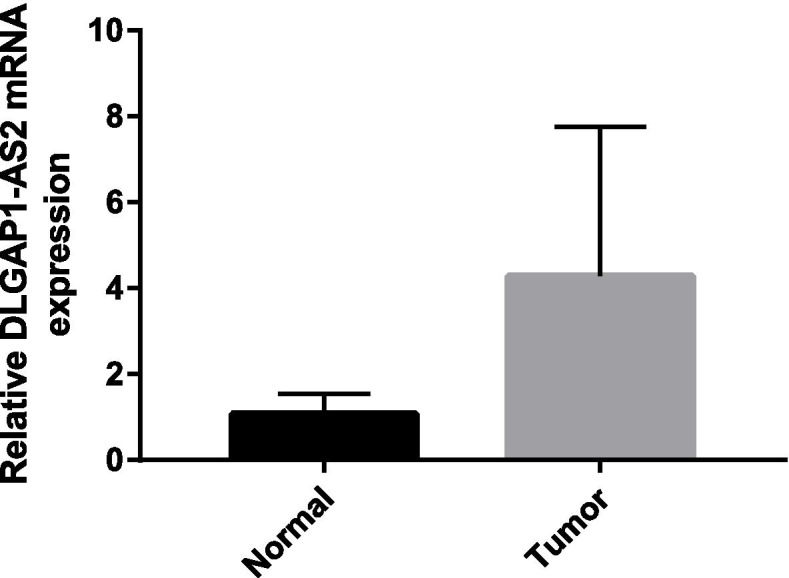
QRT-PCR analysis of DLGAP1-AS2 levels in OS tissues and adjacent normal tissues

The m6A quantitative experiment showed that the m6A level of RNA in human osteosarcoma cell lines (MG63,143B) was higher than that in human osteoblast cell line(hFOB1.19) (*P* < 0.05) (Fig. 10). The qRT-PCR analysis results showed that DLGAP1-AS2 expression increased in tumor tissues (*P* = 0.07) (Fig. 11). The *p* value > 0.05 may be caused by insufficient tissue samples. Although the *P* value> 0.05, the expression level of DLGAP1-AS2 in each pair of tissues was higher in the tumor group than in the normal group.

## Discussion

Our study investigated the prognostic significance of m6A-related lncRNAs in 84 OS patients from the TARGET dataset. We identified a total of 111 m6A-related lncRNA in our study, and eight m6A-related lncRNAs were selected by conducting a univariate Cox analysis. We then performed a KEGG analysis and a GO analysis according to the cluster assignments.. Six m6A-related lncRNAs (RP11-286E11.1, LINC01426, AC010127.3, DLGAP1-AS2, RP4-657D16.3, and AC002398.11) were identified in a LASSO Cox analysis as the most suitable risk score model for prognosis. The application value of the model was tested with ROC curve analysis and KM survival curves. In addition, a univariate and multivariate Cox regression analysis also confirmed the validity of the model.

According to GO analysis, the m6A-related lncRNAs are enriched in histone H3-K27 methylation, pericentric heterochromatin, and phosphatase activator activity. Histone H3 mutations have been found to play a role in a variety of cancers, such as paediatric brain tumors [22]. Penin et al. reported that pericentric heterochromatin of chromosome 9 was the primary target of HSF1 in both normal and tumor heat-shocked cells [23]. Zheng et al. found that LINC00174 promotes the metastasis and growth of OS through upregulating slingshot protein phosphatase 2 expression [24]. A KEGG analysis found that the m6A-related lncRNAs are enriched in the mTOR pathway. Mickymaray et al. found that rhaponticin can inhibit OS growth by inhibiting the PI3K-Akt-mTOR pathway [25]. Liu et al. reported that miR-140 inhibits OS development by influencing ubiquitin-specific protease 22 and promoting p21 expression [26].

A number of studies have suggested that m6A modification may play a regulatory role in the development of cancer. M6A-related could serve as new therapeutic targets and prognostic biomarkers for OS [27]. Miao et al. found that METTL3 regulates m6A levels of LEF1 and activates the Wnt/β-Catenin signaling pathway to promote OS progression [28]. Zhou et al. demonstrated that METTL3 acts as an oncogene in OS by regulating ATAD2 [29]. Liu et al. reported that METTL14 overexpression promotes OS cell apoptosis and inhibits tumor progression by activating caspase3 [30]. Chen et al. reported that WTAP promotes OS growth and metastasis by inhibiting HMBOX1 expression [31]. Yuan et al. found that ALKBH5 suppresses OS progression by inhibiting the pre-miR-181b-1/YAP signaling axis [32]. However, the role of m6A-related lncRNAs in OS is still unclear. We found that our risk score model built with six m6A-related lncRNAs could accurately predict the prognosis of OS. Liu et al. found that LINC01426 facilitates the progression and stemness in lung adenocarcinoma [19]. In addition, LINC01426 accelerates glioblastoma progression by regulating miR-345-3p/VAMP8 signaling axis [33]. Liu et al. reported DLGAP1-AS2 is associated with poor prognosis in cholangiocarcinoma [34]. In hepatocellular carcinoma cell, DLGAP1-AS2 knockdown inhibits cell metastasis by regulating miR-154-5p methylation [21]. We also discovered upregulated levels of DLGAP1-AS2 and m6A methylation in osteosarcoma tissues/cells compared with normal tissues/osteoblasts cells. Among the six m6A-related lncRNAs used to construct the risk prognosis model, several genes have been reported to be associated with tumor prognosis such as respiratory system tumors and digestive system tumors [35, 36]. However, no research has reported the relationship between OS and these genes or how the lncRNAs interact with m6A-related genes. Therefore, the aim of our study is to identify lncRNAs associated with OS prognosis and provide new targets to prevent poor OS prognosis. A limitation is that we have not further researched the role of m6A-related lncRNAs in osteosarcoma through cell and in vivo studies. Using the dataset downloaded from TRAGET, we constructed a risk score prognosis model of m6A-related lncRNAs. By dividing all samples into test groups and training groups, we verified the value of the model. However, the role of m6A-related lncRNAs in osteosarcoma needs to be further researched by cell and in vivo studies.

## Data Availability

All data are fully available without restriction. In this study, publicly available datasets were analyzed. This data can be found here: Therapeutically Applicable Research to Generate Effective Treatments (TARGET, https://ocg.cancer.gov/programs/target).
